# Supplementary figures and images for: SNP Markers and Evaluation of Duplicate Holdings of Brassica oleracea in Two European Genebanks
Source: Plants (Basel). 2020 Jul 22;9(8):925. doi: 10.3390/plants9080925 (PMC7465924; doi:10.3390/plants9080925)

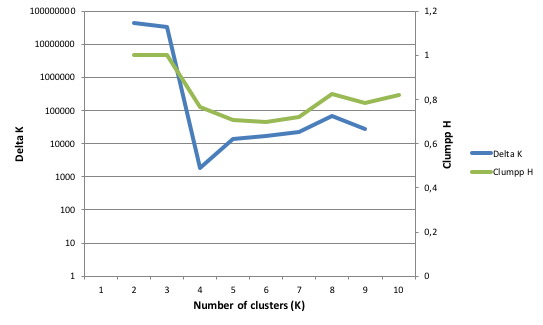

Supplement: Supplementary file 1 [file plants-09-00925-s001.zip › FigS1.tiff]

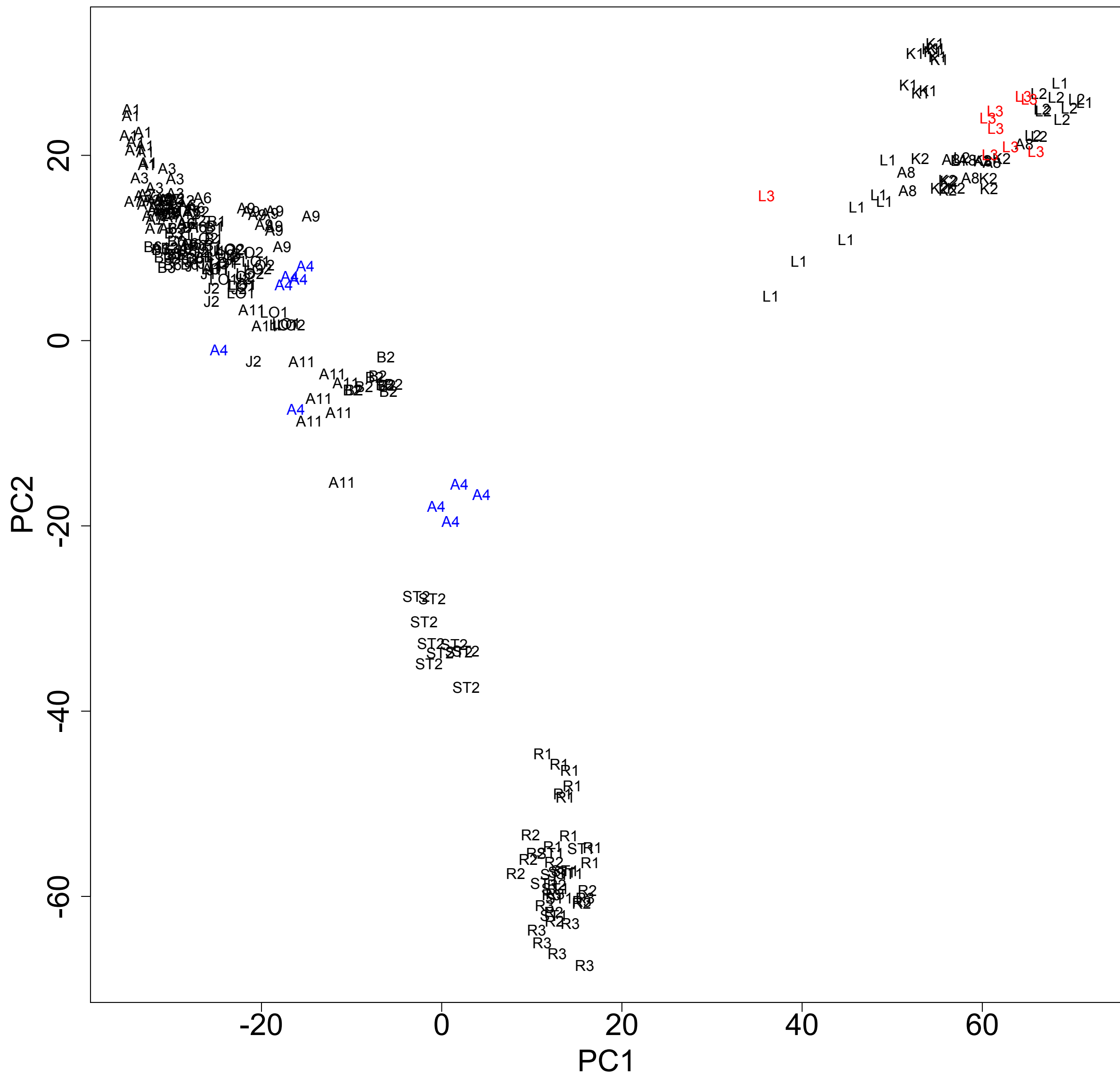

Supplement: Supplementary file 1 [file plants-09-00925-s001.zip › FigS2.pdf]

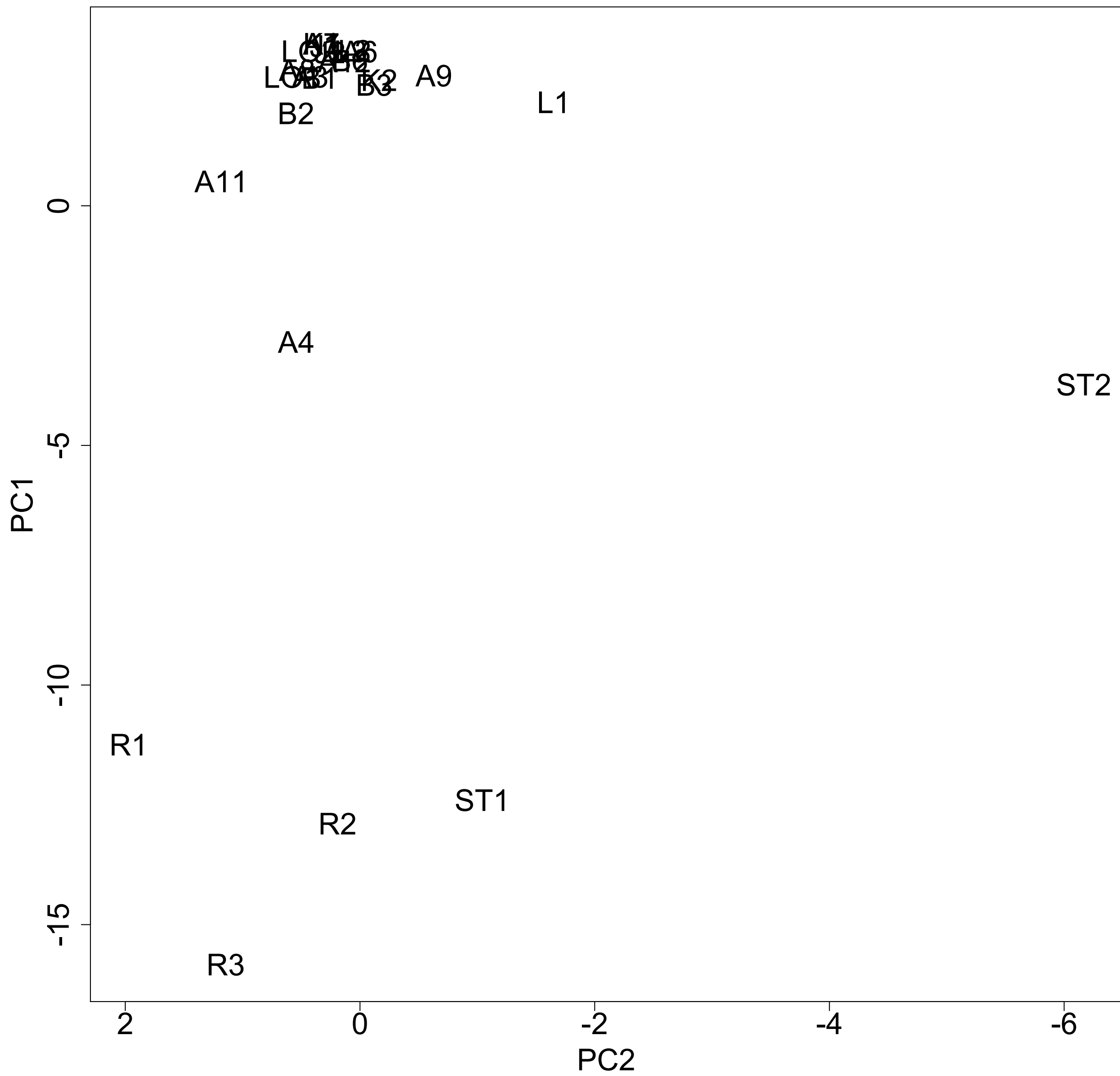

Supplement: Supplementary file 1 [file plants-09-00925-s001.zip › FigS4b.pdf]

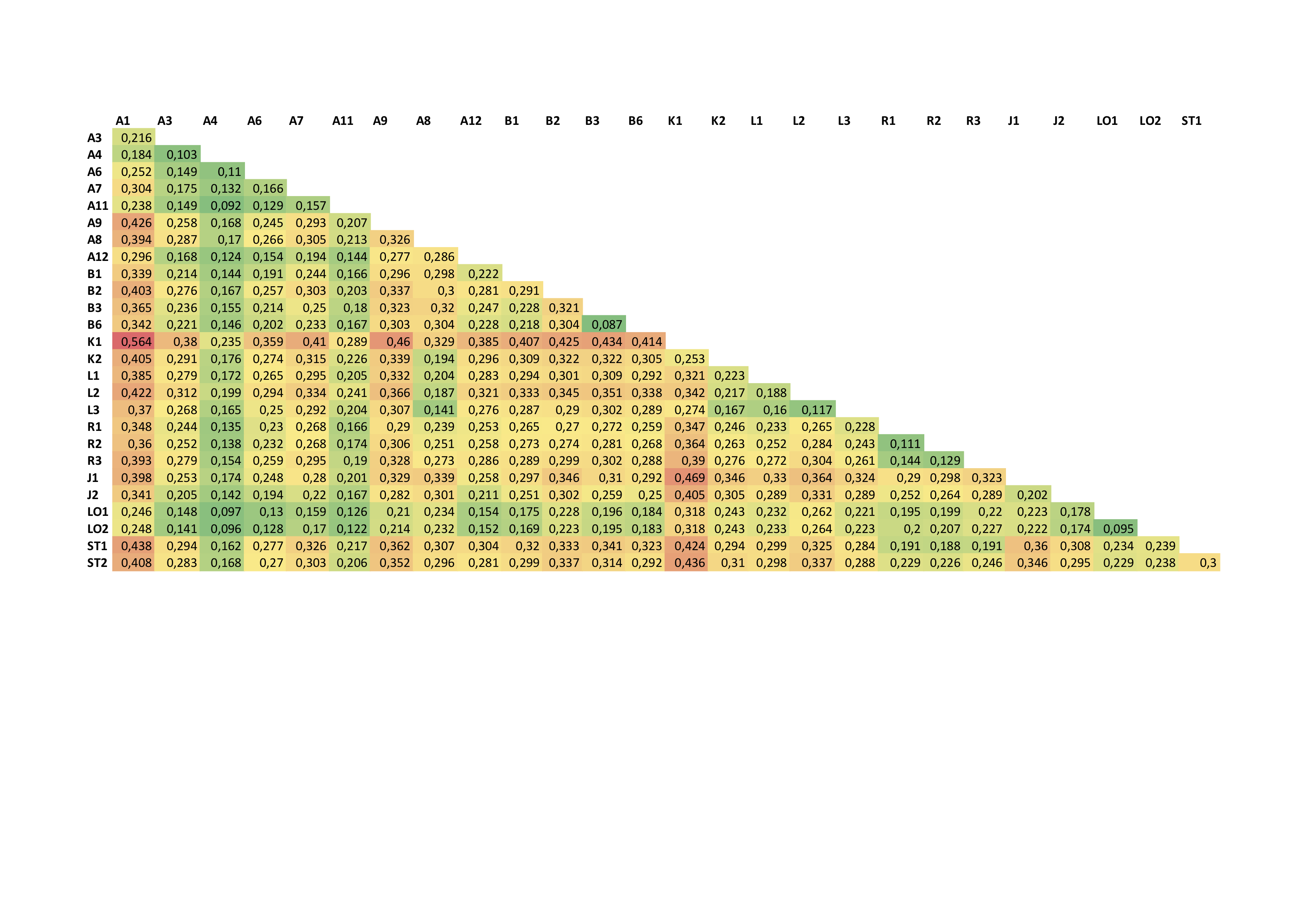

Supplement: Supplementary file 1 [file plants-09-00925-s001.zip › tableS2_FST.tiff]

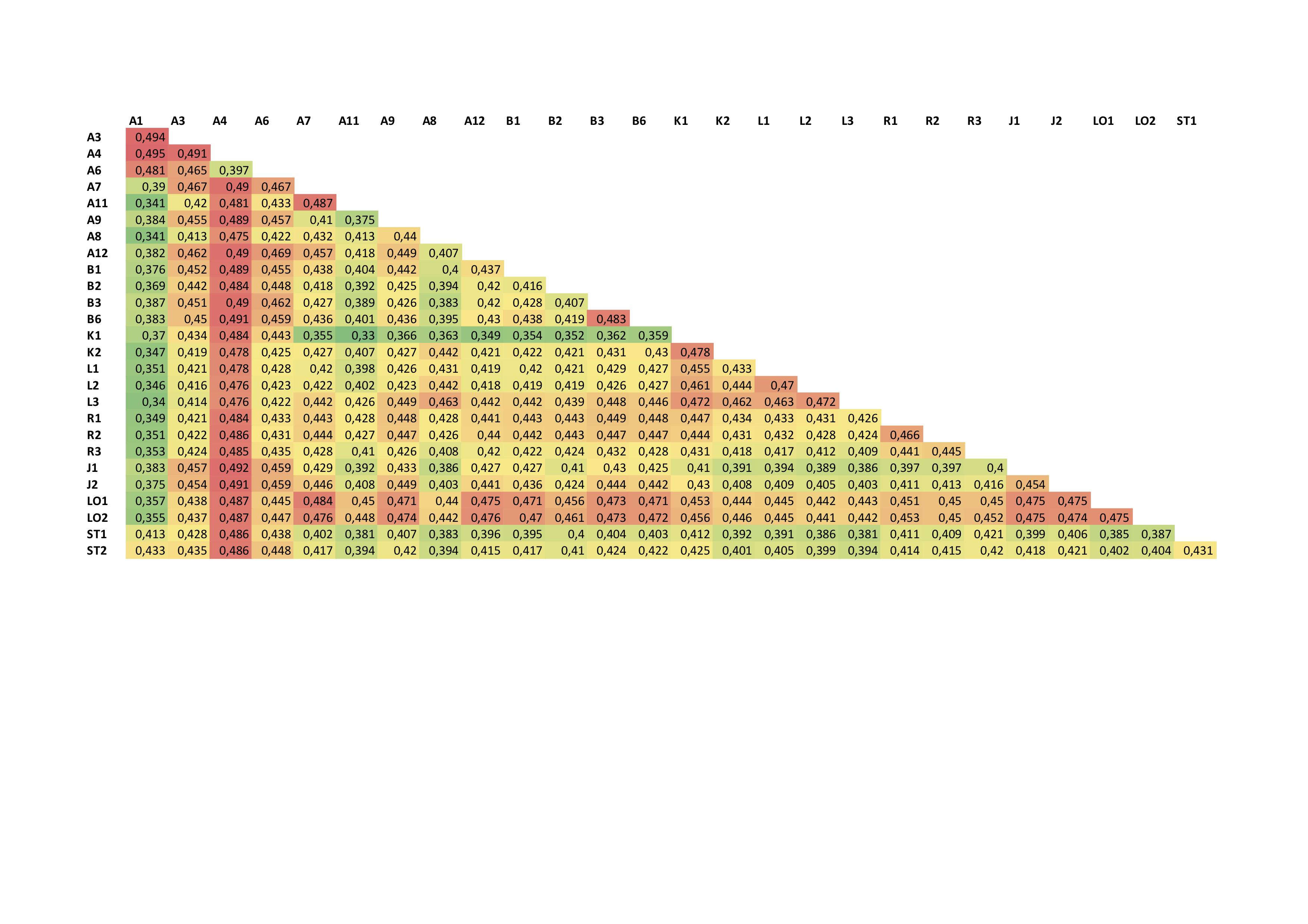

Supplement: Supplementary file 1 [file plants-09-00925-s001.zip › tableS3.tiff]
