# Supplementary material for: SNP Markers and Evaluation of Duplicate Holdings of Brassica oleracea in Two European Genebanks
Source: Plants (Basel). 2020 Jul 22;9(8):925. doi: 10.3390/plants9080925 (PMC7465924; doi:10.3390/plants9080925)
Supplement: Supplementary file 1 [file FigS4a.pdf › FigS4a.pdf]

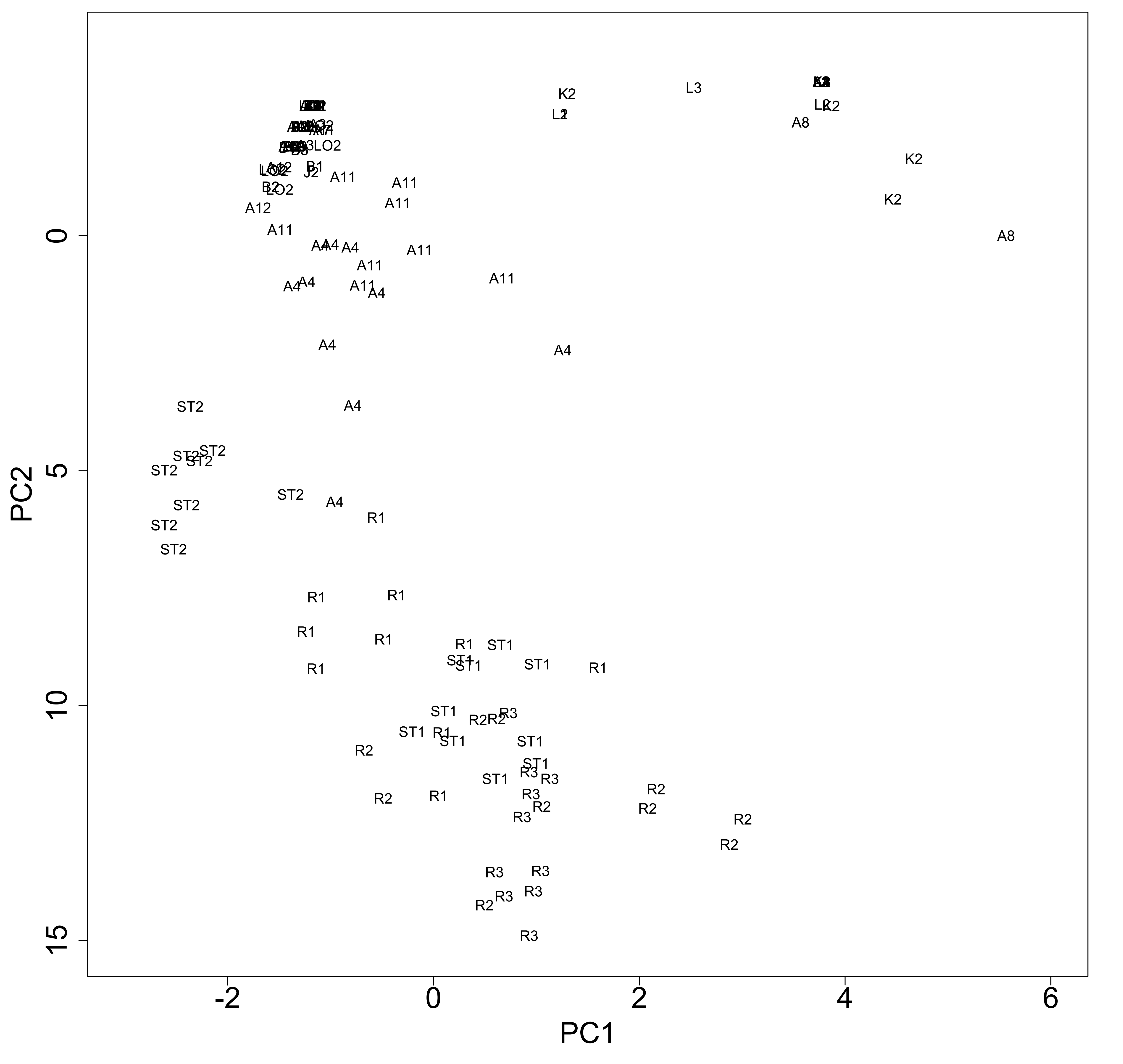
PC1
PC2
-2
0
2
4
6
0
5
10
15
